# Expanding the Genetic Code for Site-Directed Spin-Labeling

**DOI:** 10.3390/ijms20020373

**Published:** 2019-01-16

**Authors:** Theresa Braun, Malte Drescher, Daniel Summerer

**Affiliations:** 1 Department of Chemistry and Konstanz Research School Chemical Biology (KoRS-CB), University of Konstanz, Universitätsstraße 10, 78457 Konstanz, Germany; theresa.braun@uni-konstanz.de; 2 Faculty of Chemistry and Chemical Biology, TU Dortmund University, Otto-Hahn-Straße 4a, 44227 Dortmund, Germany

**Keywords:** noncanonical amino acids, bioorthogonal chemistry, spin labeling, protein conformation, EPR spectroscopy, macromolecular dynamics

## Abstract

Site-directed spin labeling (SDSL) in combination with electron paramagnetic resonance (EPR) spectroscopy enables studies of the structure, dynamics, and interactions of proteins in the noncrystalline state. The scope and analytical value of SDSL–EPR experiments crucially depends on the employed labeling strategy, with key aspects being labeling chemoselectivity and biocompatibility, as well as stability and spectroscopic properties of the resulting label. The use of genetically encoded noncanonical amino acids (ncAA) is an emerging strategy for SDSL that holds great promise for providing excellent chemoselectivity and potential for experiments in complex biological environments such as living cells. We here give a focused overview of recent advancements in this field and discuss their potentials and challenges for advancing SDSL–EPR studies.

## 1. Introduction

Electron paramagnetic resonance (EPR) spectroscopy is a powerful tool to elucidate the structure, dynamics and interactions of proteins as a basis of their physiological function. When combined with site-directed spin labeling (SDSL) [1], EPR measurements can be conducted with otherwise diamagnetic proteins and offer high sensitivity and low background compared to nuclear magnetic resonance studies. Continuous wave (cw) EPR is thereby applied to monitor the mobility of paramagnetic centers, and it provides information on the chemical environment and structural properties of the incorporation site. Moreover, double electron-electron resonance (DEER) experiments can reveal distance distributions between two paramagnetic centers in a protein, and by the width of the distributions deliver information on the conformational equilibria of the protein.

Key aspects of any SDSL–EPR study with proteins are the strategy used for incorporating the spin label (typically a nitroxide radical or gadolinium (Gd(III)) ion) into the sites of interest, as well as the chemical and structural properties of the label itself [2]. The former determines chemoselectivity and labeling degree, and in the case of labeling canonical amino acids or specific peptide motifs may prevent studying proteins in their natural state, owing to the need for removing and/or introducing such labeling sites. Moreover, chemoselectivity is key to the application of SDSL–EPR in complex biological environments such as studying endogenous proteins in in-cell measurements. Here, the high abundance of off-target labeling sites requires bioorthogonal strategies, or limits studies to the introduction of exogenous, prelabeled proteins into the cells of interest. Similarly, insufficient rates of the employed conjugation reaction can prevent effective labeling of proteins under the highly dilute conditions typically encountered in cells. On the other hand, the design of the employed label is equally important for applications in biological systems, since the stability of the newly established linker or of the paramagnetic center may be incompatible. Moreover, high conformational freedom of spin labels by the use of extensively long and/or flexible linkers can prevent gaining information on the conformational flexibility of a protein.

**Figure 1:**
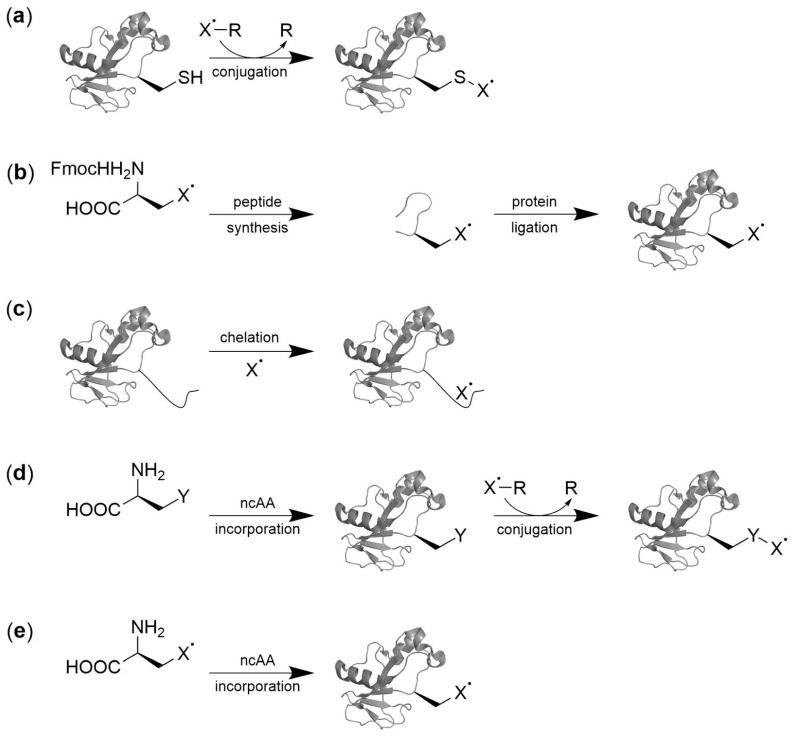
Protein SDSL strategies. Paramagnetic moieties represented by X**˙**: (**a**) Canonical AA/Cysteine labeling, (**b**) solid phase peptide synthesis followed by protein ligation, (**c**) chelation by genetically encoded peptide tags, (**d**) conjugation of ncAA with side chain Y with labeling molecule X**˙**-R, and (**e**) direct encoding of paramagnetic ncAA.

Chemoselective labeling of canonical amino acids is currently the most widely employed SDSL approach (Figure 1a). Cysteine residues are the preferred targets because of their low abundance as well as unique nucleophilicity and redox behavior [2]. Typically, labels are installed via disulfide bonds using the standard methanethiosulfonate spin label (MTSL) or via thioether bonds formed by 1,4-addition with maleimide labels, in both cases offering high chemoselectivity in the context of the target protein in vitro. However, interference with natural protein function may arise from the requirement to remove naturally occurring cysteines, and to install cysteines at sites of interest by site-directed mutagenesis. Moreover, the high number of sulfhydryl functions in cells and the low redox–stability of disulfide bonds represent hurdles for in-cell SDSL. Alternative approaches involve peptide ligation strategies (Figure 1b, [3]) or the use of chelating protein tags (Figure 1c [4,5,6,7]) that overcome some of the limitations of cysteine labeling, but in turn have limitations in terms of the incorporation site, technical ease, or the extent of the introduced structural changes into the protein under study (for comprehensive reviews covering these strategies see references [2,8,9]).

The use of genetically encoded noncanonical amino acids (ncAA) [10] is an emerging strategy in SDSL that offers a number of unique advantages for EPR studies in complex biological environments [8]. In this approach, an orthogonal pair of an aminoacyl-tRNA synthetase (aaRS) and a nonsense suppressor tRNA is introduced into the expression host. The target protein gene is engineered to contain a nonsense codon (such as the amber codon, UAG) at the position of interest, allowing co-translational incorporation of the ncAA. The ncAA can thereby either contain a paramagnetic center itself or provide a reactive handle for the introduction of the center by a bioorthogonal conjugation reaction (Figure 1d,e). Both approaches have potential to label and study proteins directly in their expression host where they are translated and processed, and thus circumvent nonphysiological conditions arising from the requirement to introduce prelabeled proteins into the cells under study.

Here, we give a brief overview of the initial studies on this topic and discuss differences between the overall labeling strategies, the employed conjugation chemistries and the spectroscopic properties of the introduced spin labels. The studies provide initial insights into the opportunities and remaining challenges of ncAA-based SDSL, and highlight their potential to further advance SDSL–EPR studies in complex biological environments.

## 2. Spin Labeling by Bioorthogonal Conjugation with Noncanonical Amino Acids

**Figure 2:**
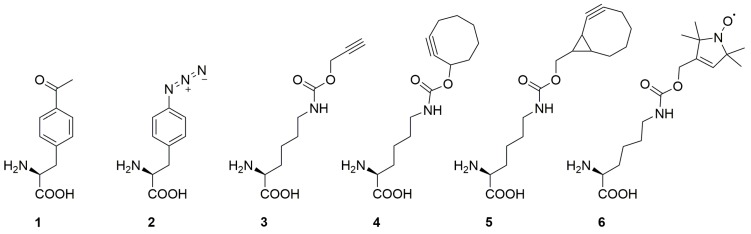
Overview of ncAA with reported use for SDSL. *p*-Acetyl-l-phenylalanine 1, *p*-azido-l-phenylalanine 2, *N*^ε^-propargyloxycarbonyl-l-lysine 3, *N*^ε^-Cyclooct-2-ynyloxycarbonyl-l-lysine 4, *N*^ε^-Bicyclo[6.1.0]non-2-yn-9-ylmethoxycarbonyl-l-lysine 5, *N*^ε^-1-oxy-2,2,5,5- tetramethylpyrroline-3-ylmethoxycarbonyl-l-lysine 6.

The introduction of spin labels into proteins via ncAA requires matching partially opposing demands in view of (1) the chemical and spectroscopic properties of the spin label, (2) the biocompatibility of the employed conjugation reactions (if the label is not directly encoded), and (3) the translation components of the expression host (Figure 2 shows an overview of ncAA previously used for spin labeling). The former demands are typically high stability of the paramagnetic center and of the linker during expression, labeling and measurement, as well as low conformational flexibility to reduce contributions of the label to the apparent conformational dynamics of the protein. Nitroxides are the most commonly employed spin labels [11], though conversion to diamagnetic products has been reported in different biological environments [12,13,14,15]. However, their stability is tunable, and depending on the overall ring size and structure as well as shielding of the nitrogen atom by differently sized substituents at the quaternary centers in α-position, significantly more stable nitroxides have been reported [16,17,18]. A limitation may arise from the increased bulkiness of reported stable nitroxides that are based on isoindoline scaffolds or bear ethyl- or even propyl-substituents in α-position. If nitroxide ncAA are to be directly genetically encoded, this has the potential to exceed the size limitations of currently available translation components, such as the employed aminoacyl-tRNA synthetase [19]. Being unaffected by reductive environments, spin labels based on paramagnetic metal cations such as the lanthanide Gd(III) in combination with chelating ligands are a promising alternative for in vivo applications [20,21,22]. Moreover, Gd(III) offers high sensitivity at high EPR frequencies and possesses a broad absorption width, making it highly suitable for DEER measurements. However, due to the bulkiness of Gd(III) labels, labeling is restricted to exposed sites of the protein. Ideal Gd(III) tags immobilize the metal ion close to the target molecule and bind the metal ion very tightly to prevent metal-mediated interactions with unspecific sites of the protein and titration effects. Though a number of metal chelating ncAA have been reported [23,24], the chelators typically employed for Gd(III) introduction are large, and no aaRS for their encoding are available, which currently limits this approach to conjugation strategies with reactive ncAA. The use of ncAA with reactive side chains for bioorthogonal conjugation generally has a high potential not only for protein labeling in presence of cysteines, but also for in vivo applications [25]. Employed reaction conditions should thereby have no impact on protein structure and stability. For in-cell labeling, all reactants have to be cell-permeable, bioorthogonal and non-toxic; less demands apply to on-cell labeling, which has been demonstrated with cysteine-labeling [26,27]. Reactions must occur at physiological pH in aqueous environments and with kinetics that match the typically highly dilute target protein concentrations in cells.

### 2.1. Condensation Reactions with Ketone Amino Acids

**Figure 3:**
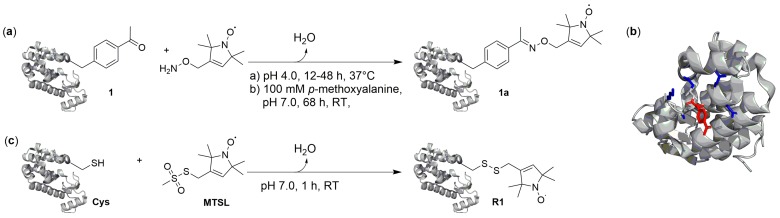
(**a**) SDSL oxime formation with ketone ncAA. Reaction of *p*-acetyl-l-phenylalanine 1 in T4-lysozyme (T4L) with a hydroxylamine nitroxide label. The nitroxide of the pyrroline group is linked via a stable C–C bonding in contrast to the disulfide linkage of R1. (**b**) Crystal structure of T4L V131*p*AcF (PDB ID 3HWL) [31]. (**c**) Labeling of cysteine in T4L with nitroxide reagent methanethiosulfonate spin label (MTSL), resulting in disulfide-linked label R1.

The ncAA *p*-acetyl-l-phenylalanine (**1** in Figure 2) bears a chemically versatile ketone group, which does not occur in canonical amino acids and readily reacts in a number of bioorthogonal condensation reactions [25]. Initially genetically encoded in *Escherichia coli* (*E. coli*) [28], this ncAA has later been encoded in *Saccharomyces cerevisiae* [29], and mammalian cells [30]. Fleissner et al. were first to demonstrate the use of for SDSL [31]. They incorporated **1** into bacteriophage T4-lysozyme (T4L) at solvent-exposed sites, purified the protein, and modified the ketone with a hydroxylamine-bearing nitroxide spin label via ketoxime-formation (Figure 3a). The comparison of crystal structures of T4L bearing 1 at position 131 (Figure 3b) with the respective wild type protein confirmed that the backbone structure was not affected by 1.

One disadvantage of this approach was the requirement for a relatively low pH = 4 or for the use of 0.1 M of *p*-methoxyaniline catalyst (at neutral pH) paired with long reactions times, which limits the potential for in-cell labeling. Nevertheless, as shown in cw EPR spectroscopy, this label is a useful sensor of local structures and conformational changes. Compared to corresponding MTSL-labeled T4L, positional uncertainties of the nitroxide were observed, arising from the additional rotatable bonds compared to R1 (Figure 3c). These were addressed by Garbuio et al. who combined the strategy with cysteine-based SDSL using a Gd(III) chelate complex. DEER measurements between nitroxide-labeled *p*-acetyl-l-phenylalanine **1** and either maleimido-monoamide 1,4,7,10-tetraazacyclododecane-1,4,7,10-tetraacetic acid (DODA) or maleimido-monoamide diethylene triamine pentaacetic acid (DTPA) loaded with Gd(III) on T4L enabled separation of intramolecular from intermolecular distance peaks in aggregations [32].

### 2.2. Copper(I)-Catalzyed Azide-Alkyne Cycloadditions (CuAAC)

Copper(I)-catalyzed azide-alkyne [3+2] cycloadditions (CuAAC) [33] have been used in different contexts of SDSL. The CuAAC proceeds under reducing conditions, which has potential to interfere with nitroxide stability (with reduction to diamagnetic hydroxylamines being a main pathway of nitroxide conversion) [34,35]. Moreover, though dependent on the used ligand, copper has cytotoxic properties [36]. Both azide- or alkyne-bearing ncAA exist that have different general properties for CuAAC-based SDSL. First, azides have been shown to undergo partial reduction in cells [37], which reduces labeling yields. Secondly, both ncAA types exist as versions derived either from tyrosine or lysine [19], with potential consequences for the flexibility of the resulting spin label.

**Figure 4:**
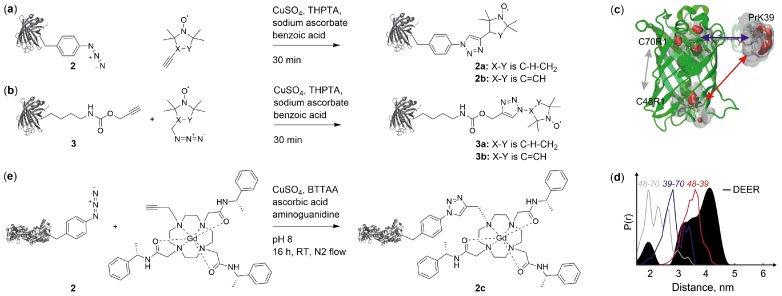
SDSL by copper catalyzed azide-alkyne cycloadditions (CuAAC). (**a**) CuAAC between ncAA 2 with azide function and a proxyl- or pyrrolin-based nitroxide label, resulting in 2a and 2b [47]. (**b**) CuAAC with ncAA 3 bearing an alkyne function and proxyl- or pyrroline-based azides, resulting in 3a or 3b [47]. (**c**) Nitroxide incorporation positions for DEER measurements with eGFP. Labels were introduced at Y39 via CuAAC using ncAA 3 (“PrK”) and at naturally occurring cysteines C48 and C70 via methanethiosulfonate spin label (MTSL) (PDB ID 4EUL [48]). (**d**) Comparison of DEER data with rotamer-based model reveals accessibility of the cysteines [47]. Measured distance distribution of eGFP Y39 3a, C70 R1 and C48 R1 (black) compared to modeled distance distributions for the respective doubly labeled protein (grey, blue, and red). (c+d) Reprinted from Orthogonal spin labeling using click chemistry for in vitro and in vivo applications, *J. Magn. Res.* **2017** *275*, 38–45 [47], Copyright (2017), with permission from Elsevier. (**e**) CuAAC between ncAA 2 with azide function and a Gd(III) label [41,42] resulting in 2c. Either tris-[(1-hydroxy-propyl-1H-1,2,3-triazol-4-yl)methyl]amine (THPTA, a+b) or 2-[4-((bis[(1-tert-butyl-1H-1,2,3-triazol-4-yl)methyl]-amino)methyl)-1H-1,2,3-triazol-1-yl]acetic acid (BTTAA, e) was used as copper-complexing ligand.

Kucher et al. successfully used CuAAC for SDSL by incorporating ncAA with either azide or alkyne functions (2 [38] and 3 [39] in Figure 2) into position Y39 of eGFP. They then reacted the proteins with respective azide or alkyne nitroxide labels (Figure 4a,b) in presence of CuSO_4_, sodium ascorbate and benzoic acid, resulting in quantitative labeling. Potential for in-cell applications was proven by detection of an EPR signal after labeling of eGFP-3a in *E. coli* cells followed by protein purification and concentration. Comparison of DEER distance measurements of eGFP asymmetrically labelled with one 3a and two R1 (Figure 3c, resulting from cysteine labeling with MTSL) including structure-based rotamer modelling revealed differences in solvent accessibility of the native cysteines of eGFP (Figure 4c,d).

In contrast to nitroxides, Gd(III) complexes offer excellent reduction stability. Abdelkader et al. incorporated *p*-azido-l-phenylalanine 2 [38] (Figure 2) with an orthogonal, polyspecific tRNA/tyrosyl-synthetase pair [40] in a cell-free reaction into the homodimeric ERp29 protein [41,42]. In absence of the release factor RF1 [43,44,45,46] to prevent premature truncation at amber stop codons and presence of purified aaRS, protein was produced in *E. coli* S30 extract to prevent partial reduction of the azide group followed by spin labeling with a Gd(III) chelate bearing an alkyne function (Figure 4e). The label was developed on the basis of 1,4,7,10-tetraazacyclododecane-1,4,7,10-tetra-acetic acid (DOTA) which is known to exhibit narrow EPR line widths and sufficiently long phase memory times.

### 2.3. Copper-Free Azide-Alkyne Cycloadditions

**Figure 5:**
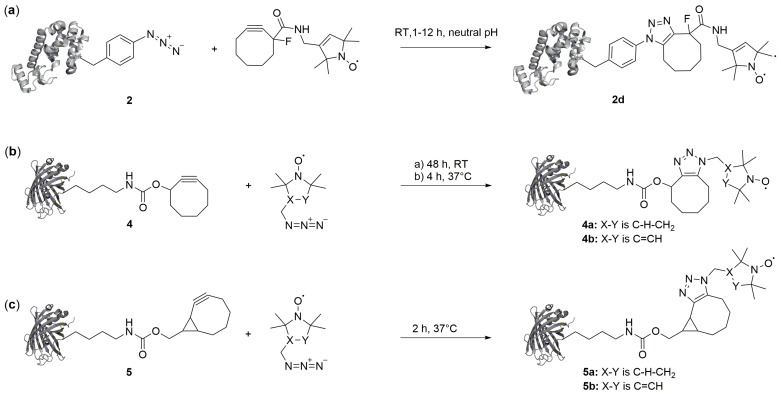
Conjugation of ncAA with nitroxide labels via strain-promoted azide–alkyne cycloadditions (SPAAC). The strained alkyne can either be part of the label reagent (**a**) or the ncAA (**b**) and (**c**).

Copper-free conjugation reactions such as strain-promoted azide–alkyne cycloadditions (SPAAC) or strain-promoted-inverse-electron-demand Diels–Alder cycloadditions (SPIEDAC) are ideally suited for cellular protein labeling because of their excellent bioorthogonality and fast reaction rates [25]. SPAAC has been used for SDSL in combination with both azide and alkyne ncAA. *p*-Azido-l-phenylalanine (2 in Figure 2) was introduced into T4L and labelled with a strained dibenzocyclooctyne nitroxide reagent (Figure 5a) [49]. However, the resulting label had a considerable size and expected flexibility, and no distance measurements were reported to elucidate the spectroscopic properties of the label. In the aforementioned study of Kucher et al., SDSL with ncAA bearing alkyne groups for SPAAC (4 [50] and 5 [51,52] in Figure 2) and two nitroxide labels with an azide group was reported (Figure 5b,c). Both ncAA were incorporated into eGFP and labeled. However, they exhibited comparably moderate labeling yields with azido-proxyl or azido-pyrroline labels, either because of insufficient reaction kinetics in case of 4 (Figure 5b) or of a suspected limited stability in case of 5 (Figure 5c) [47].

## 3. Direct Encoding of Spin Labeled Noncanonical Amino Acids

EPR and in particular in-cell DEER distance measurements in combination with SDSL can provide insights into the structural dynamics of proteins directly in cells and in a virtually background-free manner. However, despite the discussed advancements of SDSL by posttranslational conjugation with canonical or noncanonical amino acids for direct in-cell EPR measurements, this approach currently requires introduction of the prelabeled proteins into the cell (or is restricted to the cell surface). This is typically achieved by microinjection [18,53], electroporation [22], or hypo-osmotic shock [20], all techniques that do not result in physiological concentrations and localizations of target proteins. Hence, the direct intracellular labeling of proteins that have been naturally translated, folded, transported and modified would enable in-cell EPR studies of significantly increased biological relevance.

**Figure 6:**
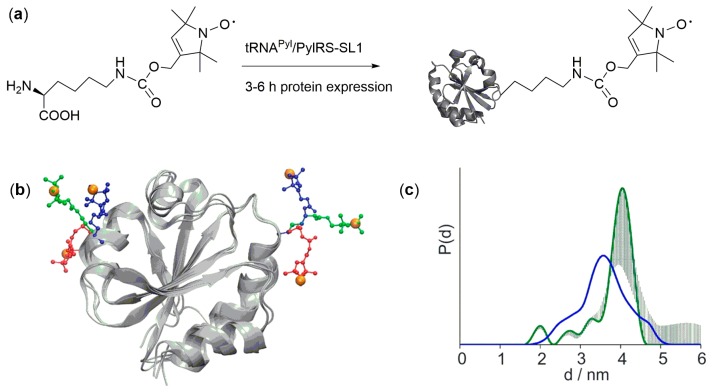
(**a**) Genetic encoding of the nitroxide ncAA **6**. (**b**) Rotamers of **6** attached to thioredoxin (pdb id 2TRX). (**c**) Distance distributions resulting from DEER experiment (green) or rotamer simulation (blue). (b+c) Reprinted with permission from EPR Distance Measurements in Native Proteins with Genetically Encoded Spin Labels, M. Schmidt, et al., *ACS ChemBiol* **2015** *10* (12), 2764–2771 [55]. Copyright (2015) American Chemical Society.

The direct encoding of nitroxide ncAA has potential to achieve this goal, since the label can be directly introduced into the (overexpressed) target protein by natural translation. The nitroxide ncAA 6 (Figure 2) was co-translationally incorporated into eGFP and thioredoxin in *E. coli* cells (Figure 6) by use of an evolved tRNA^Pyl^/PylRS-SL1 pair [54]. As a very basic experiment, singly labeled thioredoxin could be selectively detected in the washed *E. coli* host cells by cw measurements. However, though sufficient labeling yields for robust cw studies was reported for both proteins and different incorporation positions, a limited stability of the employed nitroxide in the reductive intracellular environment was still observed. This result went along with low modulation depths in DEER measurements for doubly labeled thioredoxin, and no in-cell DEER experiment was reported. Nevertheless, an experimental comparison of 6 and the MTSL-derived label R1 by DEER revealed similar widths of the distance distributions, indicating that this lysine-derived ncAA is a useful probe for DEER measurements [55,56]. Moreover, rotamer libraries were established, enabling simulations of distance distributions obtained with 6 (Figure 6b,c).

## 4. Future Directions

Some of the conceptual advantages of ncAA-based in vitro SDSL over traditional in vitro cysteine labeling have already become apparent in a number of studies, in particular the ability to study cysteine-containing proteins in their natural state. Nevertheless, for a wider acceptance of this technique, newly introduced ncAA-based approaches have to be complementary or competitive in view of cysteine labeling. This applies to labeling yields, conformational flexibility of the resulting label, and compatibility of the labeling conditions with label integrity and protein function. The most exciting perspective of ncAA-based SDSL is certainly in-cell SDSL–EPR, a quickly-growing field that offers a variety of attractive properties not covered by other methods for in-cell structural analysis. For example, compared to in-cell nuclear magnetic resonance studies, less structural information is offered by DEER but it provides a higher sensitivity and has less limitations in terms of the size of the protein(s) under study. Compared to FRET studies, DEER offers a far lower sensitivity, but also has far less background, and absolute distance distributions between the labels rather than relative distance differences can be obtained.

Key bottlenecks of SDSL that have to be overcome for effective in-cell studies are labeling bioorthogonality and biocompatibility, stability of the paramagnetic center, and kinetics of the labeling reaction, if applied. At the current state, two main strategies seem to have a particular potential for sufficiently advancing SDSL. First, a number of rapid and highly bioorthogonal conjugation chemistries are available in the context of ncAA that have been demonstrated for fluorescence labeling in various cellular and protein contexts, but are underexplored for SDSL. This includes the use of SPIEDAC with tetrazine-bearing labels or ncAA. Here, the particular spectroscopic requirements for probes used in DEER experiments (such as high rigidity) may trigger the development of alternative alkyne and alkene ncAA that are not derived from lysine. Alternatively, the direct encoding of spin labeled ncAA has potential for simple in-cell SDSL and DEER experiments, but the stability of paramagnetic ncAA has to be further improved. This may involve advanced nitroxide ncAA designs or the encoding of Gd(III)-chelating ncAA, in both cases requiring the potentially challenging design of respective aaRS. Such developments will give decisive impetus to in-cell protein SDSL–EPR studies and provide a new access to understanding protein structures, dynamics and interactions in their natural environment.

## Abbreviations

aaRS	Aminoacyl-tRNA synthetase
BTTAA	2-[4-((bis[(1-tert-butyl-1H-1,2,3-triazol-4-yl)methyl]- amino)methyl)-1H-1,2,3-triazol-1-yl]acetic acid
CuAAC	Copper(I)-catalzyed azide-alkyne cycloaddition
cw	Continuous wave
DEER	Double electron-electron resonance
DODA	1,4,7,10-tetraazacyclododecane-1,4,7,10-tetraacetic acid
DOTA	1,4,7,10-tetraazacyclododecane-1,4,7,10-tetra-acetic acid
DTPA	Diethylene triamine pentaacetic acid
EPR	Electron paramagnetic resonance
Gd(III)	Gadolinium
MTSL	Methanethiosulfonate spin label
ncAA	Noncanonical amino acids
SDSL	Site-directed spin labeling
SPAAC	Strain-promoted azide-alkyne click reaction
SPIEDAC	Strain-promoted-inverse-electron-demand Diels–Alder cycloadditions
T4L	T4-lysozyme
THPTA	Tris- [(1-hydroxy-propyl-1H-1,2,3-triazol-4-yl)methyl]amine
