# Correction: Braun, T., et al. Expanding the Genetic Code for Site-Directed Spin-Labeling. *Int. J. Mol. Sci.* 2019, *20*, 373

**DOI:** 10.3390/ijms22041574

**Published:** 2021-02-04

**Authors:** Theresa Braun, Malte Drescher, Daniel Summerer

**Affiliations:** 1 Department of Chemistry and Konstanz Research School Chemical Biology (KoRS-CB), University of Konstanz, Universitätsstraße 10, 78457 Konstanz, Germany; theresa.braun@uni-konstanz.de; 2 Faculty of Chemistry and Chemical Biology, TU Dortmund University, Otto-Hahn-Straße 4a, 44227 Dortmund, Germany

The authors wish to make the following two corrections to this paper [1]:

1. In Figures 2 and 5, a false structure of the noncanonical amino acid *N*^ε^-Bicyclo[6.1.0]non-2-yn-9-ylmethoxycarbonyl-l-lysine 5 has been shown. The corrected Figure 2 should be replaced with the following figure (Figure 1), the cor-rected Figure 5 should be replaced with the following figure (Figure 2).

2. The authors wish to add the grant number “ERC-CoG EPICODE to D.S., Grant Nr. 723863” to the Acknowledgments section.

The authors would like to apologize for any inconvenience caused to the readers by these changes.

## Figures and Tables

**Figure 1:**
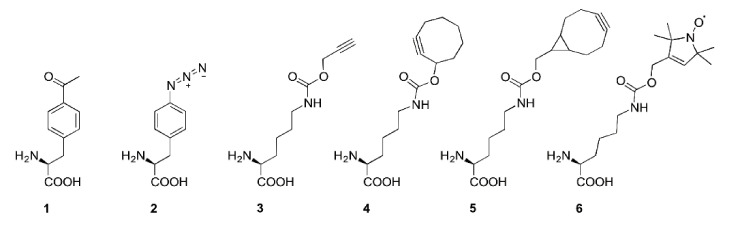
Overview of ncAA with reported use for SDSL. *p*-Acetyl-l-phenylalanine 1, *p*-azido-l-phenylalanine 2, *N*^ε^-propargyloxycarbonyl-l-lysine 3, *N*^ε^-Cyclooct-2-ynyloxycarbonyl-l-lysine 4, *N*^ε^-Bicyclo[6.1.0]non-2-yn-9-ylmethoxycarbonyl-l-lysine 5, *N*^ε^-1-oxy-2,2,5,5-tetramethylpyrroline-3-ylmethoxycarbonyl-l-lysine 6.

**Figure 2:**
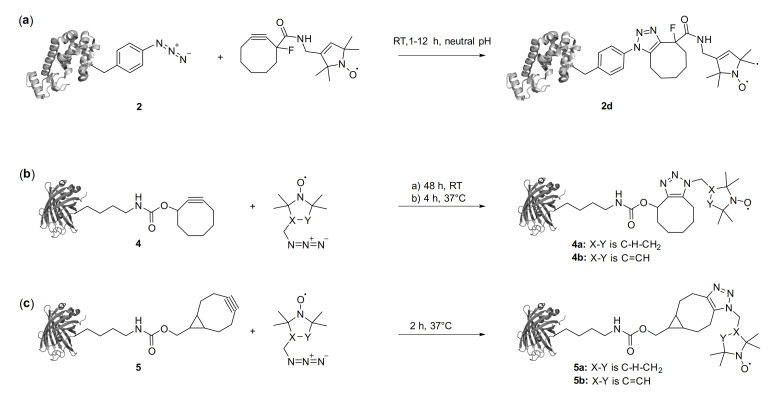
Conjugation of ncAA with nitroxide labels via strain-promoted azide–alkyne cycloadditions (SPAAC). The strained alkyne can either be part of the label reagent (**a**) or the ncAA (**b**) and (**c**).
